# Natriuretic Peptides and Blood Pressure Homeostasis: Implications for MANP, a Novel Guanylyl Cyclase a Receptor Activator for Hypertension

**DOI:** 10.3389/fphys.2021.815796

**Published:** 2022-02-11

**Authors:** Valentina Cannone, John C. Burnett

**Affiliations:** ^1^Cardiorenal Research Laboratory, Department of Cardiovascular Medicine, Mayo Clinic, Rochester, MN, United States; ^2^Department of Medicine and Surgery, University of Parma, Parma, Italy; ^3^Department of Physiology and Biomedical Engineering, Mayo Clinic, Rochester, MN, United States

**Keywords:** peptide, cGMP, receptors, genetic variation, blood pressure, natriuretic peptides, hypertension, MANP

## Abstract

The heart serves as an endocrine organ producing the hormones atrial natriuretic peptide (ANP) and b-type natriuretic peptide (BNP) which *via* the guanylyl cyclase A (GC-A) receptor and the second messenger cGMP participate in blood pressure homeostasis under physiologic conditions. Genetic models of the ANP gene or the GCA receptor together with genomic medicine have solidified the concept that both cardiac hormones are fundamental for blood pressure homeostasis and when deficient or disrupted they may contribute to human hypertension. Advances in peptide engineering have led to novel peptide therapeutics including the ANP-analog MANP for human hypertension. Most importantly a first in human study of MANP in essential hypertension has demonstrated its unique properties of aldosterone suppression and blood pressure reduction. Physiology and pharmacology ultimately lead us to innovative peptide-based therapeutics to reduce the burden of cardiovascular disease.

## Introduction

Hypertension still represents the leading cause of death worldwide. Importantly, ranking number two in cause of death is metabolic disease including diabetes and obesity both of which are predisposing and coexisting factors for hypertensive disease (Foreman et al., 2018). Much progress has been made over the last decades in developing novel therapies for hypertension both from the perspective of drugs as well as devices. Nonetheless there is increasing percentage of hypertensive patients worldwide who have uncontrolled blood pressure which cannot be attributed to non-compliance (Muntner et al., 2020). Indeed, the consensus is emerging that hypertension is a growing healthcare burden with rising morbidity and mortality. This challenge is highlighted in a report from the National Institutes of Health Working Group in Hypertension which called for new research regarding mechanisms and novel therapies especially in the setting of difficult to control hypertension such as resistant hypertension (Sigmund et al., 2020). Here, in this Review we will highlight the physiology of the heart as an endocrine organ producing the two important blood pressure lowering hormones atrial natriuretic peptide (ANP) and b-type natriuretic peptide (BNP). Key to their biology is their common molecular target, the particulate guanylyl cyclase receptor A (GC-A), and the second messenger cyclic guanosine monophosphate (cGMP) which mediates widespread cardiovascular, renal and endocrine biological actions (Kuhn, 2016). We will also highlight the importance of human genetic population studies which built upon previous genetic animal models focused on the ANP and GC-A pathway. Indeed, these human population studies have been key in laying the foundation for innovative therapeutics targeting the GC-A/cGMP system. We also summarize key advances in designer natriuretic peptides being developed to target GC-A for the treatment of hypertension.

## Blood Pressure and the Cardiac Hormones: Insights From the Laboratory and Genetic Variants in the General Population

The work of DeBold et al. (1981) launched the ever-expanding research area of the heart as an endocrine organ. The seminal observation of DeBold was that a substance from atrial myocardium of rats when injected into living animals reduced blood pressure and increased sodium excretion. Later studies elucidated that the substance was a peptide, which we defined as atrial natriuretic peptide (ANP), and subsequently its gene natriuretic peptide precursor A (NPPA) was identified. Further studies from the laboratory of Ferid Murad reported that the molecular target of ANP was membrane bound GC-A which activated cGMP (Waldman et al., 1984).

In two separate laboratories, mouse models were created to delete the ANP or the GC-A receptor genes (Lopez et al., 1995; Oliver et al., 1997). Both murine models validated that disruption of GC-A/cGMP pathway resulted in hypertension. Thus, it was established the concept that the heart is an endocrine organ which *via* a hormonal mechanism regulates blood pressure under physiologic conditions. Various studies involving additional genetic mice models reinforced the notion of cardiac hormones as blood pressure regulators. Further investigations also revealed that beyond blood pressure, GC-A protects the cardiomyocyte from hypertrophy (Holtwick et al., 2003). Importantly, a second cardiac hormone, BNP, which also binds to GC-A and activates cGMP, was later discovered. Like the ANP knockout mouse model, deletion of the BNP gene (NPPB) in rats resulted in hypertension and end organ damage, while BNP gene delivery chronically increased BNP in spontaneously hypertensive rats, reduced blood pressure and prevented organ dysfunction (Cataliotti et al., 2011; Holditch et al., 2015). These seminal laboratory studies cemented the important role of these two cardiac hormones in blood pressure regulation and in the pathophysiology of hypertension.

There was a seminal jump from the mouse to the human with the landmark study by Newton-Cheh et al. (2009). These investigators employed genomic medicine in large populations recruited in Northern Europe and North America and sought to identify NPPA and NPPB common genetic variants that were associated with circulating levels of natriuretic peptides and blood pressure. Importantly, the NPPA genetic variant rs5068 was shown to be associated with an increase in circulating levels of ANP and a small but significant reduction in blood pressure. Most importantly, this genetic variation, which was related to a lifelong increase in ANP levels, reduced the risk of hypertension underscoring the seminal role for this cardiac hormone in blood pressure homeostasis.

The Mayo Clinic is located in the Olmsted County, in southeastern Minnesota. Historically, Olmsted County has been inhabited mostly by Caucasians of Northern European ancestry. The county is unique from the standpoint that residents and families remain in the area for generations and are optimal for epidemiologic studies of human health. The Rochester Epidemiology Project is a longstanding National Institutes of Health supported study which takes advantage of the fact that almost all of the residents of Olmsted County take their care at the Mayo Clinic. Thus, medical histories, which are well preserved and maintained within the Mayo Clinic, are readily available with informed consent to elucidate the natural history of human disease and population phenotypes (Rocca et al., 2012). Cannone et al. (2011) sought to validate the observation of Newton-Chen that the ANP gene variant rs5068 was related with blood pressure and they further moved beyond investigating not only the cardiovascular but also the metabolic phenotype associated with rs5068 genotypes. The scientific background was provided by previous *in vitro* and *in vivo* studies together with human studies demonstrating that ANP and BNP induce lipolysis and browning of white adipocytes (Goetze et al., 2020). Importantly, Cannone et al. (2013) showed that rs5068 was associated with higher circulating levels of ANP and lower systolic blood pressure but also lower prevalence of obesity and metabolic syndrome. Other key findings included the relationship with higher HDL levels and reduced risk for myocardial infarction. Validation studies in a rural community in Sicily confirmed similar findings. In a follow up study in the Multi-Ethnic Study in Atherosclerosis (MESA) cohort, which includes African Americans without cardiovascular disease in the United States, Cannone et al. (2017) reported that rs5068 was associated with lower risk of obesity and metabolic syndrome but no association was found with hypertension. These observations might be related to the well documented lower levels of circulating ANP and BNP in African Americans, such deficiency may also explain the higher risk for cardiovascular disease in this ethnicity (Gupta et al., 2015).

The BNP gene NPPB is located adjacent to the ANP gene NPPA on chromosome one. The BNP genetic variant rs198389, which is a functional variant in the promoter region of the gene resulting in higher circulating BNP levels, and related clinical phenotype have been extensively studied. In a population study including African Americans and whites from the Atherosclerosis Risk in Communities (ARIC) study, Seidelmann et al. (2017) investigated phenotype and cardiovascular outcomes associated with rs198389 over two decades. The investigators reported that the minor allele had a frequency of approximately 40% and was associated with 41% higher levels of N-terminal–proBNP and, importantly, lower systolic and diastolic blood pressure, use of antihypertensive medications as well as prevalence of hypertension while lifespan was increased.

Cannone and colleagues extended studies on rs198389 focusing on a population with cardiovascular risk factors. Specifically, these investigators utilized the landmark St. Vincent Screening to Prevent Heart Failure Study (Stop-Heart Failure Study), which investigated the efficacy of using BNP based screening in combination with collaborative care between primary care physicians and cardiovascular specialists in the prevention of new onset heart failure and left ventricular dysfunction (LVD) (Ledwidge et al., 2013). Importantly, the trial demonstrated that a clinical approach focused on BNP screening and cooperative care reduced the risk to develop LVD and heart failure.

Therefore, Cannone et al. (2021) genotyped the Stop Heart Failure cohort for rs198389, assessed cardiovascular phenotype along with circulating BNP levels and performed a follow up analysis to assess risk of LVD using echocardiography as well as risk for adverse multiple cardiovascular outcomes. Key findings were that among subjects at risk for heart failure (Stage A and B heart failure) the BNP genetic variant rs198389, which is associated with higher circulating levels of BNP, was also associated with lower risk of hypertension, new onset of LVD and major adverse cardiovascular events. The authors concluded that the data support the role of BNP genetic testing in such a population as well as BNP or GC-A activating therapies for the prevention of hypertension and heart failure.

Thus, this later study went beyond a simple biomarker investigation with relevance to precision medicine (Lanfear and Luzum, 2021). The study of a single genetic variant might be considered simplistic but provides proof of concept regarding the biology of the specific gene product, which, in this case, is represented by the GC-A activating hormone BNP. This study, as well as the others referenced above, support the concept that in the presence of a relative ANP or BNP deficiency, one could pursue natriuretic peptide elevating strategies to prevent adverse cardiovascular outcomes and optimally control blood pressure.

## MANP–A Novel ANP Analog Targeting Guanylyl Cyclase a Receptor: A Therapeutic Opportunity for Hypertension

Investigations of ANP and BNP since the discovery ANP by DeBold (1981) have resulted in thousands of publications elucidating mechanisms of action supporting therapeutic potential especially in hypertension (Volpe et al., 2014; Rubattu et al., 2019). Central to the therapeutic opportunity of ANP and BNP are their physiologic properties mediated by GC-A/cGMP. These include anti-hypertrophic and anti-fibrotic actions in the heart and kidney, natriuresis and diuresis, endothelial protection, suppression of renin release and aldosterone synthesis and secretion and lipolysis and browning of adipocytes. However, as an anti-hypertensive therapeutic, ANP and BNP are limited by their relative instability in the circulation requiring that they be administered intravenously. Nonetheless, in human studies intravenous infusion of ANP in both normotensive and patients with hypertension reduces blood pressure (Biollaz et al., 1986; Janssen et al., 1989).

Advances in peptide engineering has led to novel designer natriuretic peptides for cardiovascular disease (Meems and Burnett, 2016). Employing such advances, we pursued strategic deletions, replacements or additions to the amino acid structures of native ANP, BNP and CNP, the latter the endogenous ligand to the other membrane bound GC receptor GC-B. The goals of designer peptide engineering have been to maintain or enhance safety, augment GC receptor activation, increase resistance to enzymatic degradation, individualize designs to specific clinical syndromes and develop novel delivery systems for chronic administration. MANP, developed at the Mayo Clinic, is a 40 amino acid peptide compared to 28 amino acids ANP and is highly resistant to enzymatic degradation by neprilysin and insulin degrading enzyme (Dickey et al., 2009; McKie et al., 2009; Ralat et al., 2011). It is also highly potent in activating GC-A. In the first *in vivo* study of MANP compared to ANP in normal canines, MANP demonstrated widespread superiority to ANP in reducing blood pressure, augmenting sodium excretion and inhibiting angiotensin II and aldosterone (McKie et al., 2009). In follow-up studies in an experimental model of hypertension produced by angiotensin II infusion, MANP was highly effective in blood pressure lowering and enhancing renal function while also reducing aldosterone (McKie et al., 2010). All of the above *in vivo* studies, however, utilized intravenous administration of MANP.

**FIGURE 1:**
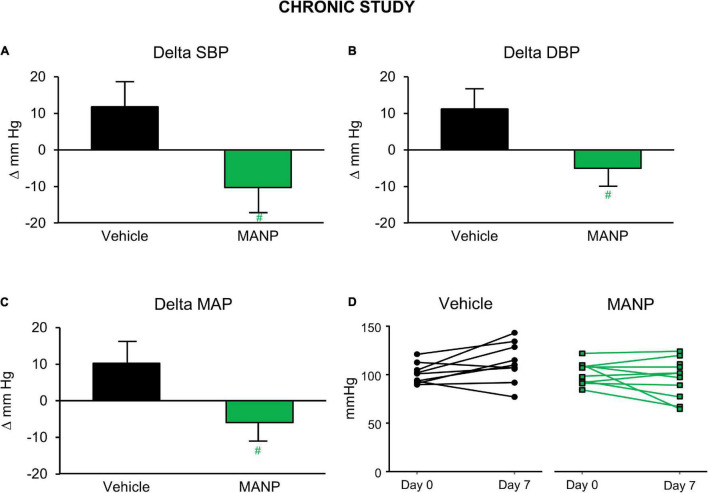
Blood pressure (BP) changes in the chronic study in salt-depletion male rats: *n* = 9 vehicle (saline) and *n* = 10 atrial natriuretic peptide analog (MANP) (3.88 mg/kg); daily subcutaneous injection for 7 days. Conscious BP was measured *via* tail-cuff method at 20 h post-injection on day 7. Delta values were recorded as changes from baseline values on day 0. Changes in systolic BP (SBP; **A**), diastolic BP (DBP; **B**), and mean arterial pressure (MAP; **C**) and individual animal MAP values on day 7 from day 0 **(D)**. #*P* > 0.05 vs. vehicle. Data are expressed as means ± SE. Unpaired *t* test was used. Reproduced with permission from the American Physiological Society (Chen et al., 2020).

**FIGURE 2:**
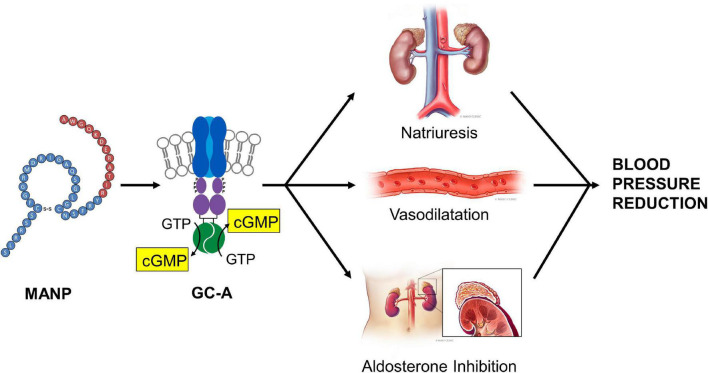
MANP Mechanism of Action: MANP, a 40 amino acid designer natriuretic peptide, activates the GC-A receptor stimulating the generation of its second messenger cGMP. After binding to GC-A in the kidney, the vasculature and the adrenal gland, MANP mediates natriuresis, vasodilatation and aldosterone inhibition. Reproduced with permission from the American Heart Association (Chen et al., 2021).

Recently, for the first time Chen et al. (2020) investigated the chronic actions of MANP on blood pressure, natriuresis, and cGMP activation in a model of hypertension in rats by subcutaneous (SQ) injection once daily thus following the paradigm of daily insulin injections used for the treatment of diabetes. A goal was to test the feasibility and effectiveness of SQ MANP delivery. Most importantly in hypertensive rats, SQ injection of MANP for 7 days induced cGMP elevation and long-term blood pressure reduction compared to vehicle (Figure 1). Thus, the study demonstrated for the first time the effectiveness of SQ administration of MANP for 7 days supporting its continued development as a novel therapeutic for hypertension. Taken together, these previous preclinical studies of MANP established MANP as a novel ANP analog which is more potent and long lasting than native ANP, and whose molecular target is GC-A resulting in enhanced production of its effector molecule cGMP. These findings support the significant therapeutic potential of MANP for the treatment of hypertension, including the challenging syndrome of resistant hypertension for which there is no FDA approved drug. Indeed, MANP possesses as a single peptide entity the pleiotropic properties of cGMP activation, natriuresis, aldosterone suppression, and BP lowering properties in preclinical studies which do not exist in any single current anti-hypertensives drug (Figure 2). These actions set MANP apart from conventional anti-hypertensive medications such as diuretics, aldosterone blockers, calcium channel blockers and renin angiotensin system antagonists (Volpe et al., 2021).

The first in human study of MANP was recently reported and performed in subjects with essential hypertension bypassing normal volunteers based upon MANP’s efficacy and safety in multiple studies in normal, hypertensive and heart failure canines and rodent models (Chen et al., 2021). The goal of this first in human study was to determine MANP’s overall safety, tolerability and plasma cGMP activating properties *via* SQ injection in hypertensive subjects who were off standard-of-care anti-hypertensive medications. The study was an open label sequential single ascending dose (SAD) design in multiple cohorts of hypertensive subjects. Three doses were employed. Natriuretic, aldosterone suppressing and BP lowering actions were assessed. Twelve patients were recruited. Inclusion criteria included (1) hypertensive subjects with SBP above 140 mm Hg; (2) on at least one anti-hypertensive medication; and (3) DBP equal to or in excess of 90 mm Hg. Exclusion criteria included (1) known hypersensitivity or allergy to MANP or its components, carperitide, other natriuretic peptides, or related compounds; (2) women who are pregnant or breast-feeding; (3) any disease or condition (medical or surgical) which, in the opinion of the investigator, might compromise the hematologic, cardiovascular, pulmonary, renal, gastrointestinal, hepatic, or central nervous system; or other conditions that may interfere with the absorption, distribution, metabolism, or excretion of study drug, or would place the subject at increased risk; (4) the presence of abnormal laboratory values considered clinically significant by the Investigator; (5) positive screen for Hepatitis B (HbsAg, Hepatitis B Surface Antigen), Hepatitis C (anti HCV, Hepatitis C Antibody), or HIV (anti-HIV 1/2); (6) received an investigational drug within a 30-day period of Visit 1; (7) consumption of alcohol within 48 h prior to dose administration or during any in-patient period; (8) a positive urine drug screen including ethanol, cocaine, THC, barbiturates, amphetamines, benzodiazepines, and opiates unless, in the opinion of the Investigator, a positive finding is as a result of a legitimate medical prescription for a valid medical condition; (9) a history (within the last 2 years) of alcohol abuse, illicit drug use, significant mental illness, physical dependence to any opioid, or any history of drug abuse or addiction; (10) a history of difficulty with donating blood or donated blood or blood products within 45 days prior to enrollment; (11) clinically significant new illness in the 1 month before screening in the opinion of the Investigator; (12) history of severe allergies (e.g., anyone with a known history of anaphylaxis to medication[s] or allergens and/or asthma requiring hospitalization); (13) history of coronary artery disease; (14) history of cerebrovascular disease; (15) history of epilepsy or other seizure disorder; (16) history of syncope; (17) history of organ transplantation; and (18) malignancy within 5 years of the screening visit (with the exception of basal cell and squamous cell skin carcinoma).

Natriuresis is a signature property of MANP *via* GC-A/cGMP. In this first in human study, investigators reported a dose response to MANP in natriuresis during the 4 h after SQ MANP. While the duration of aldosterone reduction was variable among the treatment cohorts, a signal for a reduction and off-response by 24 h was clearly observed, thus, extending to the human the aldosterone suppressing actions of GC-A activation previously reported by *in vitro* and in animal studies (Atarashi et al., 1985; Ito et al., 2003; McKie et al., 2009). Most importantly, both systolic and diastolic blood pressures were reduced with a peak action at 12 h post-dose. In addition, there was target engagement as plasma cGMP was increased. Thus, MANP induces unique reno-adrenal responses in hypertensive subjects combining natriuretic actions with the favorable action of aldosterone suppression.

**FIGURE 3:**
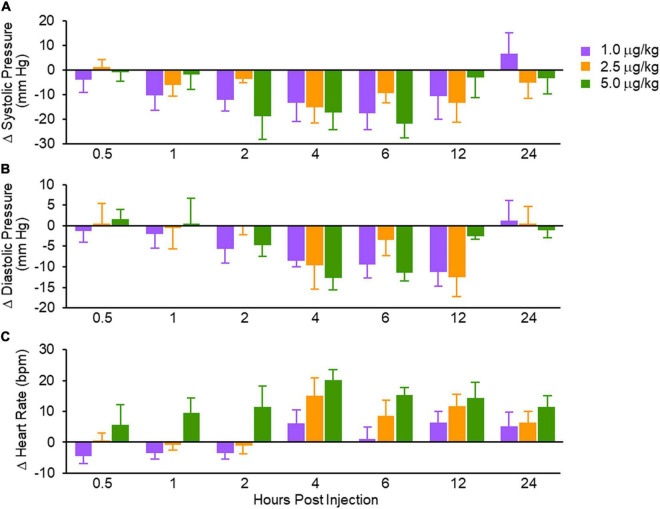
Absolute changes in systolic blood pressure **(A)**; diastolic blood pressure **(B)** and heart rate **(C)**. Values are mean ± SEM. Reproduced with permission from the American Heart Association (Chen et al., 2021).

The seminal observation was that SQ administration of MANP at three different doses reduced BP over the 24-h period of observation (Figure 3). This BP effect peaked between 2 and 12 h but there was no clear dose response. Thus, SQ doses from 1 to 5 μg per kilogram may have already achieved a maximal effect requiring follow up studies to explore lower doses. Importantly, this clinical study established that intravenous administration of a natriuretic peptide may be overcome with SQ administration as is widely used for insulin and GLP-1 analogs.

A principal goal of this first in human study was safety. No major adverse events were observed. No ECG changes in any of the subjects during the 24 h of observation were reported and there were no local reactions at the injection sites.

Thus, the first in human study of MANP in essential hypertension is transformational in hypertension therapeutics and establishes that treatment with the designer ANP analog MANP engages the GC-A receptor, activates cGMP, reduces BP, enhances sodium excretion and suppresses aldosterone. Further, MANP can be administered safely and effectively SQ and is well tolerated.

The clinical development of MANP is to target resistant hypertension which remains a huge public health burden and for which there is not approved drug. A Phase 1 placebo control 3-day multiple ascending dose study in resistant hypertension has been completed and data are being analyzed from 20 patients. In this completed Phase 1 trial in resistant hypertension, MANP was administered on top of three or more antihypertensive drugs which included diuretics, angiotensin converting enzyme inhibitors (ACEi), angiotensin A1 (AT1) receptor blockers and calcium channel blockers. Thus, data will be available on the blood pressure reducing actions of MANP on top of conventional anti-hypertensive agents. A new expanded clinical trial has been initiated in 40 patients with resistant hypertension (20 Caucasians and 20 African Americans) which is placebo controlled and with multiple ascending doses. MANP is administered once daily for 5 days.

The current delivery method for MANP is by subcutaneous injection once daily. As advances in peptide therapeutics in cardiovascular and metabolic disease accelerate, SQ delivery is emerging as with GLP-1 agonists for diabetes and PKC9 inhibitors for hypercholesterolemia. Nonetheless, research and development of MANP also includes the development of oral delivery of MANP. Here, advances in oral delivery of peptides such as BNP and GLP-1 agonists have also been reported supporting the feasibility of this exciting delivery strategy for peptides such as MANP (Cataliotti et al., 2008; Drucker, 2020).

It should be stated that several questions remain to be addressed as MANP studies continue. These include establishing the ability of MANP to chronically reduce blood pressure and activate the cGMP pathway thus not having drug tolerance. In addition, the role of gender in responsiveness to MANP also needs to be established as sex-based differences in circulating ANP and BNP exist with plasma ANP and BNP being higher in females. Further, the potential modulating action of ANP and BNP genetic variants, which are associated with variations in circulating levels of the two hormones, should also be investigated in regard to blood pressure response to MANP. While ANP as an intravenous drug for heart failure has proved successful in Japan, the use of BNP (nesiritide) and urodilatin (ularitide) in heart failure has been disappointing. This may be related to excessively high doses which are hypotensive as well as their use only as acute drugs as opposed to chronic therapy at a lower dosage. Indeed, hypertension may be the most physiologic target for natriuretic peptide therapy based upon the evidence of a relative deficiency of ANP and BNP in human hypertension and blood pressure reduction being the signature pharmacologic action of MANP.

Another drug breakthrough that involves the natriuretic peptides system by augmenting their circulating levels is sacubitril/valsartan. Sacubitril inhibits the enzyme neprilysin (NEP) which is widely expressed in the human body and degrades ANP and BNP. Valsartan serves as an AT1 blocker. Sacubitril/valsartan was approved for heart failure based upon an improvement in mortality in the pivotal PARADIGM HF trial (McMurray et al., 2014). Ruilope et al. (2010) reported the effectiveness of sacubitril/valsartan compared to sacubitril or valsartan alone in human hypertension in which sacubitril/valsartan was superior to either NEP inhibition or AT1 blockade. Both MANP and sacubitril/valsartan may be valuable novel therapies for hypertension involving molecular mechanisms which are not activated in conventional anti-hypertensives. However, through NEP inhibition, not only is the degradation of ANP and BNP inhibited, but also vasoconstrictor peptides such as angiotensin II (ANG II), which is also degraded by NEP, may increase in the circulation. Indeed, in heart failure, ANG II is higher in the circulation with sacubitril/valsartan than with either an AT1 blocker or ACEi (Pavo et al., 2016). Thus, MANP emerges as a selective GC-A activator devoid of non-GC-A actions and can also be administered SQ determining higher levels of ANP which exceeds that which can be achieved with NEP inhibition. A high priority is to understand populations which best respond to these two drugs in hypertension in further studies in hypertension.

Since the discovery of ANP by Adolfo DeBold that established the heart as an endocrine organ, the field of natriuretic peptides is again accelerating. It is not unusual that it has taken four decades to also establish the therapeutic potential natriuretic peptides and testing in humans. That may be the usual cycle of drug development. This includes designer peptides such as MANP and novel small molecules as sacubitril/valsartan which may inhibit the breakdown of endogenous ANP and BNP and boost their circulating levels (Ruilope et al., 2010; Chen et al., 2021). Perhaps also employing precision medicine to identify patients with ANP and/or BNP deficiency along with cardiac hormone replacement therapeutics might be the future successful strategy to reduce the burden of cardiovascular disease.

## Data Availability Statement

The original contributions presented in the study are included in the article/supplementary material, further inquiries can be directed to the corresponding author.

## Author Contributions

Both authors conceived the outline of the manuscript, wrote the first and final draft, contributed to the article, and approved the submitted version. JB designed the figures. VC finalized the references.

## Conflict of Interest

Mayo Clinic has licensed MANP to E-STAR BIO and JB is the inventor of MANP. The remaining author declares that the research was conducted in the absence of any commercial or financial relationships that could be construed as a potential conflict of interest.

## Publisher’s Note

All claims expressed in this article are solely those of the authors and do not necessarily represent those of their affiliated organizations, or those of the publisher, the editors and the reviewers. Any product that may be evaluated in this article, or claim that may be made by its manufacturer, is not guaranteed or endorsed by the publisher.
